# A Policy for the New Year

**Published:** 1921-01-01

**Authors:** 


### The Hospital

The Workers' Newspaper of Administrative Medicine and Institutional Life, Administration, National Insurance and Health.

No. 1804, Vol. LXIX. SATURDAY, JANUARY 1, 1921. PRICE TWOPENCE

### A POLICY FOR THE NEW YEAR.

In spite of the gloomy prognostications with which the past year began, the voluntary system is still with us. The pessimists would have been very much dismayed had their own prophecy been fulfilled. Indeed, it may be said that the position is stronger, for, to give these pessimists their due, the needs of the moment are better understood than they were a year since, and something has been done to provide for them. It was our duty to prevent a panic, so far as we were able, but this did not hinder us from encouraging attempts, whenever made, to improve the general position. The result of these attempts is happily evident in the improved position of the Sunday Fund, in the measure of success which has attended the efforts of Mr. Philip Inman and Mr. R. J. Coles, and in the improved organisation of the British Hospitals Association. The task for 1921 is to make all these and kindred efforts still more fruitful, so that in the New Year we may not only reap the harvest sown, however humbly, last year, but extend the area of financial cultivation. For encouraging as the above endeavours have been, they are only beginnings, and the movement for the wider collection of small sums must be much extended if a recurrence of debt and panic is to be prevented.

We hope, then, that further co-ordination of societies interested in the voluntary system may take place, and that an agreed policy may be formed in regard to the insurance societies, and to the organisation of funds from shops and offices, at present unaffiliated to the Saturday Funds. Only last week we were able to record some hopeful work in Birmingham, where the Hospital Council seems at last to have set to work, and to have begun to provide not merely organisation, but suggestions of policy such as those of Sir Gilbert Barling, which we mentioned. Efforts like these are potentially far more fruitful than such methods as charges towards maintenance, even though these may become the general order of the day. The fine improvement shown by the Hospital Sunday Fund is an instance of the vitality of familiar sources of income, and yet how many, till the result was known, had actually despaired that Hospital Sunday would ever rise much above its former self! Exactly the same applies to employers' contribution schemes, which are still only in their infancy, especially in London, always the laggard in respects like these. Even in Leeds, where the new scheme for employers was more carefully devised than anywhere, the scheme is far from complete, but that which it has already gained is only an earnest of future possibilities if the original impetus be not allowed to wane.

If the energies centred in the Government as the *deus ex machina* of hospital difficulties had been solely devoted to the voluntary system's own native resources, the progress lately shown would have been twice what it is. For you cannot make the most of voluntary effort if your secret hopes reside in the opposite camp, and the real lesson of the past year has been to expose the old fallacy of such hopes and to make the most of traditional sources of income. In the correspondence, lately published, between Lord Knutsford and ourselves he told us, with a readiness which we appreciate, how he had been spending his time, and, as we had already indicated, this had been occupied in the rooms of Ministers. May we not inquire of him now if the results have justified these visits? May we not ask also if he would not be carrying fewer anxieties had he not spent less time with Dr Addison and more with the heads of banks and businesses or insurance offices? The word of these latter gentlemen, at all events, takes effect. It is time that we suggest to Lord Knutsford the harder task; for he is the last to shrink from hard work when the voluntary hospitals need him. For example, fruitful as Mr. Coles' recent plans have been, they have not been free from the objection that they might conflict with others already adopted. We regretted that Mr. Coles did not answer our queries in his recent letter. But could not he and Lord Knutsford help each other by discussing how Mr. Coles' plan would work if patients' contributions are to continue? This is only a humble instance, but all beginnings are humble, and our main point is to urge all parties not to plough the sands, whence no harvest can be reaped but disappointment, and to concentrate rather on familiar ground, which joint effort can make productive. That is the first policy that we urge for the coming year.

